# Implementation fidelity to a behavioral diabetes prevention intervention in two New York City safety net primary care practices

**DOI:** 10.1186/s12889-023-15477-2

**Published:** 2023-03-28

**Authors:** Avni Gupta, Jiyuan Hu, Shengnan Huang, Laura Diaz, Radhika Gore, Natalie Levy, Michael Bergman, Michael Tanner, Scott E. Sherman, Nadia Islam, Mark D. Schwartz

**Affiliations:** 1 School of Global Public Health, New York University, 708 Broadway, New York, NY 10003 USA; 2 Department of Population Health, NYU Grossman School of Medicine, 180 Madison Ave 2F Rm 222, New York, NY 10016 USA; 3 Department of Population Health, NYU Grossman School of Medicine, 180 Madison Ave, 2Nd Floor, New York, NY 10016 USA; 4 Department of Population Health, NYU Grossman School of Medicine, 180 Madison Ave, 9-43A, New York, NY 10016 USA; 5 Department of Population Health, NYU Grossman School of Medicine, 180 Madison Ave, New York, NY 10016 USA; 6 Department of Medicine, NYU Grossman School of Medicine, 462 First Avenue, Area 2d, New York, NY 10016 USA; 7 Department of Medicine, NYU Grossman School of Medicine, 423 East 23Rd Street, Room 16049C, New York, NY 10010 USA; 8 VA New York Harbor Healthcare System, 423 East 23Rd Street, Room 16049C, New York, NY 10010 USA; 9 Department of Medicine, NYU Grossman School of Medicine, 462 1St Ave, New York, NY 10016 USA; 10 Department of Population Health, NYU Grossman School of Medicine, 180 Madison Avenue, New York, NY 10016 USA; 11 VA New York Harbor Healthcare System, 180 Madison Avenue, New York, NY 10016 USA; 12 Department of Population Health, NYU Grossman School of Medicine, 180 Madison Avenue, Suite 955, New York, NY 10016 USA; 13 VA New York Harbor Healthcare System, 180 Madison Avenue, Suite 955, New York, NY 10016 USA

**Keywords:** Implementation fidelity, Process evaluation, Community health workers, Diabetes Prevention, Lifestyle intervention

## Abstract

**Background:**

It is critical to assess implementation fidelity of evidence-based interventions and factors moderating fidelity, to understand the reasons for their success or failure. However, fidelity and fidelity moderators are seldom systematically reported. The study objective was to conduct a concurrent implementation fidelity evaluation and examine fidelity moderators of CHORD (Community Health Outreach to Reduce Diabetes), a pragmatic, cluster-randomized, controlled trial to test the impact of a Community Health Workers (CHW)-led health coaching intervention to prevent incident type 2 Diabetes Mellitus in New York (NY).

**Methods:**

We applied the Conceptual Framework for Implementation Fidelity to assess implementation fidelity and factors moderating it across the four core intervention components: patient goal setting, education topic coaching, primary care (PC) visits, and referrals to address social determinants of health (SDH), using descriptive statistics and regression models. PC patients with prediabetes receiving care from safety-net patient-centered medical homes (PCMHs) at either, VA NY Harbor or at Bellevue Hospital (BH) were eligible to be randomized into the CHW-led CHORD intervention or usual care. Among 559 patients randomized and enrolled in the intervention group, 79.4% completed the intake survey and were included in the analytic sample for fidelity assessment. Fidelity was measured as coverage, content adherence and frequency of each core component, and the moderators assessed were implementation site and patient activation measure.

**Results:**

Content adherence was high for three components with nearly 80.0% of patients setting ≥ 1 goal, having ≥ 1 PC visit and receiving ≥ 1 education session. Only 45.0% patients received ≥ 1 SDH referral. After adjusting for patient gender, language, race, ethnicity, and age, the implementation site moderated adherence to goal setting (77.4% BH vs. 87.7% VA), educational coaching (78.9% BH vs. 88.3% VA), number of successful CHW-patient encounters (6 BH vs 4 VA) and percent of patients receiving all four components (41.1% BH vs. 25.7% VA).

**Conclusions:**

The fidelity to the four CHORD intervention components differed between the two implementation sites, demonstrating the challenges in implementing complex evidence-based interventions in different settings. Our findings underscore the importance of measuring implementation fidelity in contextualizing the outcomes of randomized trials of complex multi-site behavioral interventions.

**Trial registration:**

The trial was registered with ClinicalTrials.gov on 30/12/2016 and the registration number is NCT03006666.

**Supplementary Information:**

The online version contains supplementary material available at 10.1186/s12889-023-15477-2.

## Background

The NationalInstitutes for Health recommends fidelity measurement in health behavior studies because without the knowledge of implementation fidelity, it may be impossible to draw correct inferences about the effectiveness of or to replicate an intervention [1]. Erroneous inferences may lead to invalid conclusions about the relationships between the intervention components and outcomes, especially in pragmatic studies of complex behavioral interventions [2, 3]. However, most pragmatic trials do not report systematic assessment of implementation fidelity and very few assess how participant and contextual factors may influence fidelity. For example, a review found that only 3.5% of complex behavioral interventions for drug abuse adequately addressed intervention fidelity to planned core intervention components [4].

CHORD (Community Health Outreach to Reduce Diabetes), launched in 2017, is a cluster-randomized, pragmatic trial that tests a Community Health Worker (CHW) driven intervention to promote healthy lifestyle changes to reduce the incidence of Type II diabetes mellitus (DM) among pre-diabetic patients cared for in two safety-net primary care sites that serve veterans, or uninsured and Medicaid populations [5]. We sought to improve the understanding of subsequent CHORD trial results, as well as to learn about how contextual factors affected implementation of the CHORD intervention, by assessing fidelity to its four core components across the study’s two participating sites.

By reporting on the fidelity of the CHORD study implementation, our study attempts to fill a gap in systematic documentation and reporting of implementation processes of complex behavioral interventions [4, 6]. In addition to clarifying causal mechanisms, assessment of implementation fidelity can provide critical information to guide future implementation of the intervention [4, 7–9]. Examining implementation fidelity is particularly necessary for multicomponent, complex behavioral interventions because of their higher likelihood of deviation from the protocol [9]. Few studies conduct concurrent fidelity assessment as opposed to retrospective. Concurrent process evaluations are important as they can capture implementation experiences in real time [9]. By building in the collection of fidelity measures and moderating factors in CHORD implementation, this study seeks to address this gap in the literature. Moreover, the study responds to the general calls for conducting quantitative evaluations of fidelity in intervention studies [9].

**Fig. 1:**
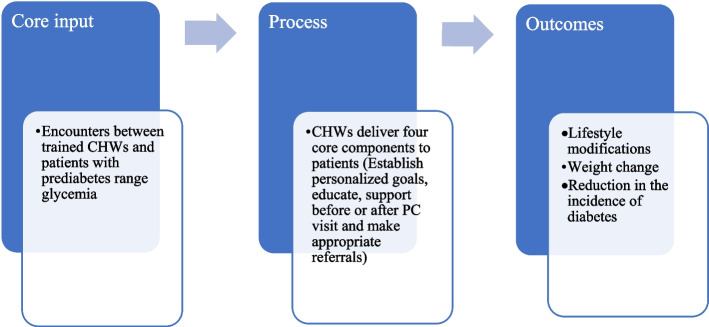
Logic Model and the Theory of Change of the CHORD Intervention

Figure 1 shows the logic model for the CHORD intervention. Evidence supports engagement of CHWs by safety net primary care practices for improving health, knowledge, behaviors and outcomes for underserved communities [10, 11]. The CHORD CHWs deliver evidence-based lifestyle interventions known to prevent DM among people with prediabetes [12–14]. CHWs are trusted members of the community who share lived experience and are familiar with community resources and norms [5, 10, 15]. Culturally congruent CHWs can bridge gaps between communities and health care systems to facilitate positive behavioral and lifestyle changes [12–14]. The evidence-based lifestyle interventions implemented in CHORD were based on guidelines from the National Diabetes Prevention Program (DPP) with a focus on healthy eating and physical activity [16]. People with prediabetes who participated in such a structured lifestyle change program reduced their risk of DM by 58% [16].

CHORD is a complex behavioral intervention, where CHWs facilitate patients’ adoption of guideline-based lifestyle changes by using four behavioral intervention components: patient goal setting; education topic coaching; facilitating primary care (PC) visits; and referrals to address social determinants of health (SDH) [17]. We assessed fidelity to these four behavioral intervention components and examined factors affecting implementation fidelity using the Conceptual Framework for Implementation Fidelity (CFIF) [6, 7]. The overarching purpose of this analysis is to inform researchers and program implementors about the level of fidelity achievable of an intervention such as CHORD in the context it was implemented.

## Methods

### Trial participants and description

The CHORD trial’s priority population was patients with prediabetes receiving care at two PC clinics, the Manhattan campus of the VA NY Harbor Healthcare System (VA), and Bellevue Hospital Center (BH) of New York City’s municipal hospital system [5]. Protocol details have been described previously [5]. This study was approved by the New York University Langone Health and the Veterans Affairs Institutional Review Boards and was registered with clinicaltrials.gov (NCT03006666) on 30/12/2016. Informed consent was obtained from all the participants in the study. All the procedures were followed in accordance with the relevant guidelines (eg. Declaration of Helsinki) along with the rest of the ethical declarations. Findings were reported in accordance with the StaRI checklist for implementation studies [18].

Briefly, all PC clinicians within each site were randomized to intervention or usual care. Eligible patients were seen by one of the study clinicians (at least 1 PC visit in the past 2 years at the VA or at least 3 PC visits in the past 2 years at BH), were between ages 18–75 years, had ≥ 1 glycosylated hemoglobin (HbA1c) between 5.7–6.4% at baseline, no prior DM diagnosis, and ability to communicate in English or Spanish. Two CHWs, members of the study team assigned to each site, consented and enrolled eligible intervention patients, who then completed an intake survey, and began a 12-month intervention during which at least monthly encounters were planned between the CHW and patient. The two CHWs at each site made up to ten attempts to call and enroll each intervention group patient. A successful encounter was defined as when the CHW was able to speak with the patient in-person or by phone, or when a letter or a text message was delivered (no evidence of failure to deliver was apparent). During these encounters, CHWs delivered one or more of four core intervention components.

### Implementation of CHORD behavioral components

In the first component, CHWs established individualized goals with each patient and completed a 6-item, Patient Activation Measure (PAM) [19, 20]. These goals were then translated into a health action plan (HAP) tailored to each patients’ goals, PAM score, and preferences. Second, using the HAP, CHWs chose among 22 educational topics organized into 5 modules (prediabetes, healthful eating, MyPlate [21] and plate portions, physical activity, and stages of change) to conduct education sessions with patients, and provide information packets. Third, CHWs called or met patients before and/or after PC visits to encourage them to discuss diabetes prevention with their clinician or to review their after-visit summaries regarding diabetes. Finally, CHWs facilitated referrals as needed to hospital or community-based programs to help support behavioral change or address identified social needs.

### CHW training and fidelity monitoring

To facilitate and standardize the implementation of the intervention, CHWs received comprehensive training and then ongoing feedback during weekly team meetings and case review sessions. To address behavioral components, CHWs completed training on coaching competencies, motivational interviewing, mental health and nutrition needs, weight management and healthy lifestyle programs, multicultural competence, diabetes and diabetes prevention, elderly and loneliness, and technical trainings on using Excel, Outlook, and REDCap (Research Electronic Data Capture).

### Data collection

**Fig. 2:**
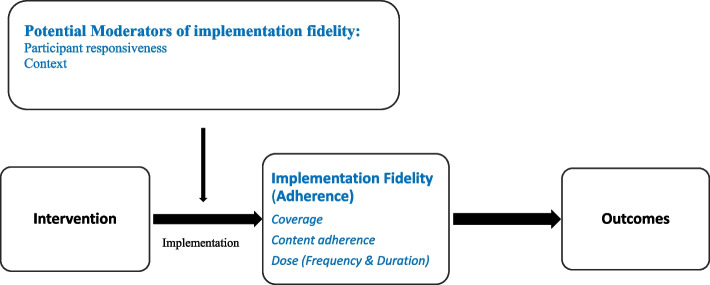
The Modified Version of the Conceptual Framework for Implementation Fidelity that guided Fidelity Assessment of the CHORD intervention (adapted from Carroll et al. and Hasson et al.)

Figure 2 shows the modified version [6] of the Conceptual Framework for Implementation Fidelity (CFIF) [7] which guided our data collection and analyses. The key indicator of implementation fidelity according to CFIF is *adherence,* defined by Consolidated Framework of Implementation Research (CFIR) as “whether a program service or intervention is being delivered as it was designed or written.” [7] We measured adherence as *coverage*(“what proportion of target group participated in the intervention”) [6], *content*(“was each of the intervention components implemented as planned”) [6], and *dosage,* which includes *duration* and *frequency*(“amount of an intervention received by participants” [7], or “were the intervention components implemented as often and for as long as planned”) [6]. We also measured two factors that could moderate fidelity: *Context*(“what factors at the political, economic, organization, and work group levels affected the implementation”) [6], and *participant responsiveness* (“how were the participants engaged with the intervention”).

Data on the CFIR implementation fidelity elements identified above were collected from the start of the CHORD trial (December 2017) until October 2019, using standardized Research Electronic Data Capture (REDCap) [22] forms completed by CHWs for each patient to record their demographics, information on outreach, enrollment, and intake, and weekly logs on encounters. Electronic Health Records (EHR) from VA and BH provided descriptive data on study participants and their healthcare utilization.

### Measures of fidelity and fidelity moderators

Table 1 shows the data sources for measures of fidelity and fidelity moderators. Coverage was the percent of patients who completed an intake and hence were able to receive the intervention. Content adherence was the percent of patients who received each of the four intervention components. Dose frequency was how much of the four components was received measured as an average or median across all intervention patients and dose duration measured for how long the intervention was delivered.

**Table 1:** Study measures and their data sources mapped to the framework constructs

Framework construct	Reported measure from the CHORD trial	Corresponding CHORD intervention core component if applicable	Data Source
**Fidelity**			
Coverage	Percent of outreached patients who were enrolled	N/A	Outreach form
	Percent of enrolled patients who completed intake	N/A	Intake form
	Percent of intake patients who completed the first core component of establishing at least one goal or a Health Action Plan^a^	1	Goal Setting Form
Content adherence	Percent of intake patients who established at least one goal or a Health Action Plan^a^	1	Goal Setting Form
	Percent of intake patients who received coaching on at least one education topic	2	Encounter Form
	Percent of intake patients who received coaching on all education modules	2	Encounter Form
	Percent of intake patients who had at least 1 PC visit	3	Electronic Health Record
	Percent of intake patients who received at least one referral	4	Encounter and Referral Forms
	Percent of intake patients who received at least one successful encounter	N/A	Encounter Form
	Percent of intake patients who received all four core components in some capacity	1–4	Goal Setting, Encounter and Referral Forms
Dose- frequency	Median number of goals established	1	Goal Setting Form
	Median number of goals completed	1	Goal Setting Form
	Median number of education sessions delivered	2	Encounter Form
	Median number of education modules discussed	2	Encounter Form
	Median number of PC visits	3	Electronic Health Record
	Median number of referrals	4	Encounter and Referral Forms
	Median number of successful encounters	N/A	Encounter Form
Dose- duration	Median duration (days) of follow-up time^b^	N/A	Encounter Form
**Moderating factors**			
Participant responsiveness	Baseline Patient Activation Measure Score (< median vs. ≥ median)	N/A	Intake Form
Context	Clinical site (VA vs. BH)	N/A	Study Form

^a^Percent of intake patients who established at least one goal or completed establishing a Health Action Plan was operationalized as a measure of two fidelity constructs – content adherence and coverage – because this component was the first component that patients were required to complete in order to proceed with other components of the intervention

^b^Duration of follow-up is the time from beginning of outreaching till the last successful encounter

Measures of hypothesized moderators:

1. *Participant responsiveness,* measured as the baseline *Patient Activation*Measure (PAM) score, which measures the extent to which patients are activated for participating in managing their health and healthcare including seeking health information and readiness to change [23–25]. According to Hasson et al., “the uptake of the intervention depends on the responsiveness of those receiving it” (page 2). [6] We hypothesized that people with higher PAM score (indicating higher activation to participate in health) will be more responsive and motivated to participate and receive the intervention.
2. *Context,* measured as the two clinical sites. As the intervention components were designed to be delivered by CHWs to patients, the clinical site was selected to capture systematic differences in the sociodemographic and economic context of *patient* populations at the two sites. It should be noted that, organizational differences between the two sites were not directly hypothesized to moderate fidelity measures of CHORD intervention which relied on individual encounters between CHWs and patients. Therefore, clinical site reflects differences in patients’ social profiles at the two sites. We did not assess the role of other specific patient characteristics that are not explained by their chosen site of healthcare.

### Analysis

Fidelity measures of coverage, content and dose were reported using applicable descriptive statistics including percentage for categorical variables or median with inter-quartile ranges (IQRs) for continuous or count variables. To evaluate fidelity moderation, we computed unadjusted *p*-values from Chi-square or Mann–Whitney U test and adjusted *p*-values from regression models that controlled for patient gender, language, race, ethnicity and age. To assess moderation by PAM score, we dichotomized the score as above or below the median score. As a sensitivity analysis, we also compared patients with a total maximal score of 24 vs. < 24. The first regression models treated PAM score as the primary independent variable, and the second set treated clinical site as the primary independent variable. Each set of models included separate models for each fidelity measure as the dependent variable. Logistic regression models were used for binary measures of coverage and content adherence (received or not received the intervention component). Linear regression models were used for fidelity measures of dosage that were continuous, and negative binomial regression models were used to model the fidelity measure of dosage that was a count variable.

Among patients who completed intake (*n*= 444), the denominator for our fidelity assessment, 32 patients (7.2%) with missing PAM scores were excluded from fidelity moderation analysis by PAM score. Chi-square test or Mann–Whitney U test, as appropriate, were used to compare two population groups—how eligible/outreached patients differed by their enrollment status and how enrolled patients differed by their intake completion status – on their gender, language, race, ethnicity, age at outreach and PAM score. All quantitative analysis was conducted in the R statistical software environment [26], and statistical significance required an alpha < 0.05.

## Results

### Fidelity measures

#### Coverage

**Fig. 3:**
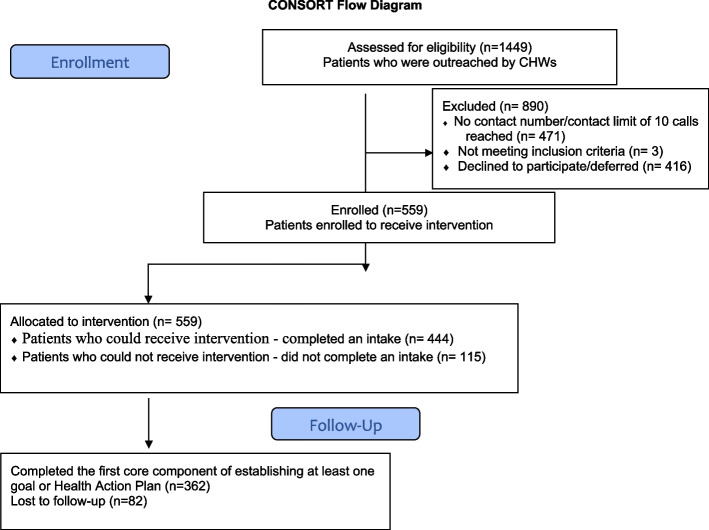
CONSORT Flow Diagram for the Intervention Arm

Among 1449 eligible patients assigned to CHWs for outreach, we excluded 471 patients with incorrect/no contact information or who could not be reached after ten phone calls, 416 who declined to participate, and another three found to be ineligible. Of the remaining 559 patients who enrolled in the intervention arm, 444 (79.4%) completed an intake survey and were eligible to receive the intervention, hence comprised the analytic sample for fidelity assessment (Fig. 3). The 559 enrolled and the 890 unenrolled patients differed in their primary language, race, ethnicity, implementation site and median age at outreach. Those who enrolled were more likely to be Spanish speaking, Black and Hispanic (Supplemental Table 1). However, among those enrolled, patients completing intake vs. those not completing intake were similar (Supplemental Table 2). Among intake patients, 362 (81.5%) established a HAP, the first core component.

**Table 2:** Implementation fidelity (Content Adherence) for each intervention component

Measure	Relevant core component	Number completing the component	% Completing the component (*n* = 444)
Percent of intake patients who established at least one goal or a Health Action Plan	1	362	81.5%
Percent of intake patients who received coaching on at least one education topic ^a^	2	367	82.7%
Percent of intake patients who received coaching on all education modules	2	106	40.0%
Percent of intake patients who had at least 1 PC visit	3	353	79.5%
Percent of intake patients who received at least one referral	4	200	45.0%
Percent of intake patients who received at least one successful encounter ^b^	N/A	354	79.7%
Percent of intake patients who received all four core components in some capacity	1–4	155	34.9%

^a^The number of patients who received coaching on at least one education topic were more than the number of patients who established at least one goal (or a Health Action Plan) because although not designed to, 5 patients received an educational coaching session before they established a goal (or a Health Action Plan)

^b^A successful encounter between a patient and a CHW was defined as an encounter (after the completion of the intake survey) where the CHW was able to speak with the patient either in-person or by phone, or when a letter or a text message was delivered (that is, when no evidence of failure to deliver was apparent). The number of patients with a successful encounter is less than the number of patients who completed at least one goal or the number of patients who received coaching on at least one education topic because although not designed to, for some patients, after a patient completed an intake, goal establishment or education sessions happened without a successful encounter

#### Content adherence

Eighty percent of patients had ≥ 1 successful encounter with the CHW (Table 2). About 80% of the patients completing an intake established ≥ 1 goal for their HAP, received coaching on ≥ 1 education topic or had ≥ 1 PC visit, indicating high content adherence to these three core components. Forty percent received coaching on all five education modules. Forty-five percent received a SDH referral, most of which were for a healthcare visit, with other referrals to facilitate healthy lifestyle, employment/workforce training, social security benefits, or mental health services. Overall, 34.9% received all four core components in some capacity.

#### Dose – frequency and duration

 Among the 362 patients who established a HAP, more than half established three goals (median 3; IQR: 2, 3), more than half completed ≥ 1 goal, and 25% completed three goals (median:1; IQR: 0, 3). The median number of education sessions delivered was 18 (IQR: 8, 33) and the median number of education modules covered was four of five (IQR: 2, 4). Median number of referrals was one (IQR: 1, 3), with 25% receiving ≥ 3. The median number of PC visits was three (IQR: 2,5). A total of 4,072 encounters with patients were documented by CHWs, of which 55.6% were successful. The median number of successful encounters per patient was five (IQR: 3, 8) (Table 3). Among them, 9.9% had 12 successful encounters. The median duration of follow-up was 411 days [IQR: 341, 446], as we extended the overall intervention period to > 12 months for some patients.

**Table 3:** Implementation fidelity (frequency and duration) for each intervention component

Measure	Relevant core component	Median [IQR]
Frequency		
Median number of goals established	1	3 [2,3]
Median number of goals completed	1	1 [0, 3]
Median number of education sessions delivered	2	18 [8,33]
Median number of education modules discussed	2	4 [2,4]
Median number of PC visits	3	3 [2,5]
Median number of referrals	4	1 [1,3]
Median number of successful encounters	N/A	5 [3,8]
Duration		
Median duration (days) of follow-up time^a^	N/A	411 [341, 446]

^a^Duration of follow-up is the time from beginning of outreaching till the last successful encounter

### Impact of moderators on fidelity measures

#### Patient activation

The median PAM score was 18 of a maximal score of 24. None of the fidelity measures were moderated by PAM score when dichotomized at the median (Supplemental Table 3) or at the highest score of 24 versus < 24 (results not presented).

#### Context

The implementation of the CHORD intervention was moderated by clinical site, with 60% of patients from BH and 40% from VA. VA patients had higher coverage and overall content adherence than BH. But a greater percentage of BH patients received coaching on all education modules and received all four core components. Three content adherence measures, including percent of patients who received ≥ 1 referral, ≥ 1 successful encounter and ≥ 1 PC visit, were similar at the two sites. Three dose-frequency measures, including median number of PC visits (4.0 VA vs. 4.0 BH), number of education modules covered (4.0 VA vs. 4.0 BH), and the number of successful encounters (4.0 VA vs. 6.0 BH), differed between the two sites (Supplemental Table 4).

## Discussion

In the CHORD study, we hypothesized that trained CHWs, through individualized goal setting, educational coaching, and facilitated referrals, would support positive lifestyle changes and prevent the onset of diabetes among patients with prediabetes. However, an intervention may not affect lifestyle change if it deviates from its protocol during implementation. In this concurrent process evaluation of the CHORD intervention, we examined implementation fidelity and fidelity moderators. Our analysis demonstrated moderate to high (depending on intervention component) rates of implementation fidelity of CHORD, and moderation by implementation site. The level of implementation fidelity achieved in this intervention is likely to be sufficient to justify inferences drawn about the impact of the intervention on the outcomes. However, with less than 100% adherence and the variability observed in these components, as is likely common in complex behavioral interventions, the analysis of CHORD outcomes will provide an opportunity to explore the relative importance and impact of each of these components, accounting for their degree of implementation fidelity. Our use of quantitative methods for fidelity assessment will allow us to use these measures in outcome data analyses to determine the role of fidelity in observed outcomes [1, 27].

We found that CHWs completed an intake with nearly 80% of the patients enrolled in the intervention arm, and three of the four core components (goal setting, education and PC visits) were delivered to nearly 80% of the patients. Even though coverage and content adherence were moderate to high, there was high variability in the dosage. While the fourth core component in our intervention, referrals for social determinants of health and promoting access to healthy lifestyle choices, was delivered to only 45% of the patients, a quarter received 3 or more referrals. As referrals were designed to be tailored to each patient’s circumstance, the dosage of referrals cannot be directly associated with outcomes as more referrals are not necessarily better. Further, we do not know if the remaining 55% patients who did not receive a referral needed one.. We strived to deliver all core components for each patient, but we had not established, a priori, thresholds for adherence for core components among study participants. Our analysis explores the extent to which we attained implementation success as measured by percent of patients for which the core components were delivered. An implementation fidelity analyses of a randomized trial delivering a complex care continuum intervention for frail elderly people in Sweden also reported high variability in the adherence to intervention components. On a 4-point scale (never, seldom, sometimes, often, always), while most components were reported to be ‘always’ delivered, several were only ‘seldom’ delivered [28]. In another study implementing a program to promote care aide involvement in formal team communications about resident care, the adherence was comparable to our study. Nearly 63% of the units participated in all three workshops and nearly 80% of the units participating were delivered the inter-team activities [9]. However, some measures were higher than our study – 93% of the units in this trial completed goal setting [9]. In another trial implementing a complex behavior change intervention to enable participants living with long-term musculoskeletal pain to improve their quality of lives, although the median score on adherence to components ranged from 1.67 to 2.00 on a scale of 0 to 2.00, overall course score of 2.00 showed 100% adherence to these components [29]. Higher fidelity in interventions such as those targeting pain can be explained by the nature of the intervention. According to CFIR, the relative advantage of the intervention plays a key role in implementation success: [30] “If users perceive a clear, unambiguous advantage in effectiveness or efficiency of the innovation, it is more likely the implementation will be successful….benefits of the innovation must be clearly visible (observable) to assess relative advantage” [31]. The benefits of our intended outcome, diabetes prevention in patients with pre-diabetes, are more intangible and hence could have reduced patients’ motivation to participate fully in the intervention components.

We assessed the role of patient motivation in our study by examining moderation of fidelity measures by PAM score. Null findings on moderation by PAM score was unexpected because patient activation measured by PAM scores has been found to be positively associated with engaging in healthy lifestyle behaviors and exhibiting readiness to change [25]. PAM score at intake might be limited in its representation of the concept of ‘participant responsiveness’ as defined in the CFIF [6, 7]. Patient activation might not be directly associated with the perception of patients about the effectiveness of an intervention in improving health, even though they value health. Finally, using an adapted, shortened version of the PAM [19, 20], may not have fully measured this construct of activation.

The pragmatic nature of the CHORD trial permitted and promoted continuous adaptations. While this improved the meaningfulness of our intervention, it resulted in deviations from the original intervention protocol, decreasing fidelity. We adapted implementation in response to ongoing developments and situations. For example, the recruitment and follow-up process changed significantly after BH adopted a new EHR system in the second year of the CHORD trial. Provider communication and follow-up was adapted to align with their work schedule. In five cases, CHWs delivered education sessions before establishing goals as an engagement strategy to demonstrate how the intervention could be useful and encourage patients to set goals. In addition, the number of patients with a successful encounter was less than the number of patients who completed at least one goal or the number of patients who received coaching on at least one education topic. This occurred because for some patients, CHWs completed intake, established goals and provided some education in the same intake visit, but were unable to follow-up with the patient after repeated attempts, resulting in patients with goals and education but few successful encounters. The median number of successful encounters was 5 and about 10% patients had 12 or more successful encounters. As the CHORD protocol planned at least monthly contacts between a CHW and patient, these findings suggest that CHWs divided their time between meeting motivated patients less frequently and checking-in more frequently with patients who perhaps had more complex social needs. All these adaptations could have impacted our fidelity. Future analysis will focus on sharing these adaptations or natural deviations in implementation of a pragmatic trial.

Our finding of moderation by the implementation site underscores the need to account for site-based differences in patient characteristics such as their social risk profiles, when implementing evidence-based interventions. Although BH and VA are both safety-net settings, they differ in terms of their patient populations.BH is the flagship of New York City’s large, municipal hospital system, and serves a diverse, multicultural and multilingual population with high numbers of poor and racial/ethnic minorities, many of whom are uninsured or on Medicaid. VA NY Harbor is smaller, and provides care to veterans (mostly older, White, male, and English speakers) and is funded federally. Variation in the complexity of patient populations have historically necessitated innovation, and the differences in fidelity at the two sites could be reflecting such adaptations by CHWs to meet the needs of their patient populations. Earlier studies have found different implementation fidelity across different organizations [32, 33], without any impact on outcome differences at patient-level [27]. These findings, including ours, suggest that an effective implementation fidelity might be based on the local organization’s conditions and therefore might require implementors to consider local adaptations when scaling-up evidence-based behavioral interventions. It is also important to note that, while our use of implementation site to measure ‘context’ reflects different healthcare ecosystems in terms of the patients served, neither of these clinical sites nor the scope of our analysis fully measures ‘context’ as conceptualized in the CFIF. A comprehensive assessment of patients’ social environments is an important consideration in a CHW-led intervention such as CHORD, which relies on patients’ achieving behavior change in the conditions of their everyday lives.

These differences in patient populations at the two sites could also explain the different participation rates at the two sites. Utilizing CHWs as a healthcare workforce is an equity-oriented approach to improving healthcare access and health outcomes among underserved populations [34]. Given their purpose, training and demographic composition (belonging to racial-ethnic minority groups), it is not surprising that the patients who enrolled in our CHW-led intervention were more likely to be Spanish speaking, Black, Hispanic, and seeking care at Bellevue hospital – these population subgroups have historically been excluded or marginalized from healthcare systems and face challenges in accessing resources to live a healthy life. Our CHWs were also either black or Hispanic, and those at the VA were veterans.

### Study limitations

First, as a pragmatic trial, the CHORD implementation did not start or end on fixed days, because CHWs maintained continued contacts with their patients. As a result, some interventions, such as referrals, were delivered outside of the intervention period. We included them in this analysis if they were recorded by CHWs. Second, fidelity measures can be intervention specific. Therefore, the measures used in this study might not directly translate to other complex interventions. Third, one important aspect of implementation is how well the participants engage with the intervention. In our study, while we measured the delivery of core components from the perspective of CHWs, we could not assess the extent to which delivered interventions were received by patients. Fourth, with two CHWs per site, differences in approach and skill by the CHWs, despite their uniform training and monitoring, may have contributed to the differences by site. Fifth, with four CHWs, we did not have enough variation in race/ethnicity to track patient enrollment by the concordance of their race/ethnicity with that of CHWs’. Sixth, we report fidelity measures across the three waves of CHORD implementation, but do not examine changes in fidelity over time. Finally, while we report on fidelity delivery, our study did not measure fidelity receipt or fidelity enactment [9], or assess qualitative aspects of fidelity, such as the quality of goal setting or the comprehensiveness of education sessions beyond the coverage of the required education modules.

### Study strengths and contributions

Our study adds to the limited literature with systematically reported concurrent evaluation of implementation processes of multicomponent complex behavioral interventions [4, 6]. Moreover, the study responds to the general calls for conducting quantitative evaluations of fidelity in intervention studies [9]. Use of real time data reported by the key implementors, the CHWs, adds to the validity of our analysis. Lastly, our study empirically tested the CFIF and found that the framework is a useful tool for conceptualizing and organizing measures of fidelity and their moderators. However, our process evaluation suggests that to standardize quantitative fidelity assessments, the field will benefit from further guidance on “how-to” quantitatively measure fidelity moderators.

## Conclusion

Our concurrent quantitative, implementation evaluation of a complex pragmatic trial to prevent diabetes in safety-net settings, found moderate-to-high adherence to the core components of the intervention, as well as moderation of several fidelity measures by implementation site, with no impact of the baseline patient-activation measure on fidelity measures. Analyses of implementation fidelity of complex interventions such as this trial, advances the field of implementation science. This implementation evaluation will inform our analyses of the study outcomes and may be useful for other researchers conducting complex behavioral interventions.

## Supplementary Information

**Additional file 1: Supplement Table 1.** Characteristics of Patients Who Were Determined Eligible for the Intervention Arm (and were Outreached by CHWs), by Their Enrollment Status. **Supplement Table 2.** Characteristics of Patients Who Were Enrolled in the Intervention Arm, by their Intake Completion Status. **Supplement Table 3.** Moderation of Fidelity Measures by PAM Score Among Intervention Patients Completing the Intake Survey*. ***Supplement Table 4.** Moderation of Fidelity Measures by Clinical Site* Among Intervention Patients Completing the Intake Survey.

## Data Availability

The datasets used and/or analyzed during the current study are not publicly available due to patient identifiers but is available from the corresponding author on reasonable request.

## Abbreviations

BH: Bellevue Hospital
CFIF: Consolidated Framework for Implementation Fidelity
CHORD: Community Health Outreach to Reduce Diabetes
CHW: Community Health Worker
DM: Type 2 Diabetes Mellitus
HAP: Health Action Plan
IQR: Inter-quartile range
NY: New York
PAM: Patient Activation Measure
PC: Primary care
REDCap: Research Electronic Data Capture
VA: Veterans Administration
