# Supplementary material for: Implementation fidelity to a behavioral diabetes prevention intervention in two New York City safety net primary care practices
Source: BMC Public Health. 2023 Mar 28;23:575. doi: 10.1186/s12889-023-15477-2 (PMC10045092; doi:10.1186/s12889-023-15477-2)
Supplement: Supplementary file 1 — Additional file 1: Supplement Table 1. Characteristics of Patients Who Were Determined Eligible for the Intervention Arm (and were Outreached by CHWs), by Their Enrollment Status. Supplement Table 2. Characteristics of Patients Who Were Enrolled in the Intervention Arm, by their Intake Completion Status. Supplement Table 3. Moderation of Fidelity Measures by PAM Score Among Intervention Patients Completing the Intake Survey. Supplement Table 4. Moderation of Fidelity Measures by Clinical Site* Among Intervention Patients Completing the Intake Survey. [file 12889_2023_15477_MOESM1_ESM.docx]

**Implementation Fidelity of a Complex Behavioral Intervention to Prevent Diabetes Mellitus in Two Safety Net Hospitalsin New York City**

Avni Gupta^1^, Jiyuan Hu^2^, Shengnan Huang^2^, Laura Diaz^2^, Radhika Gore^2^, Natalie Levy^3,4^, Michael Bergman^3,5^, Michael Tanner^3^, Scott E Sherman^2,5^, Nadia Islam^2^, Mark D Schwartz^2,5^

^1^School of Global Public Health, New York University, New York, NY

^2^Department of Population Health, NYU Langone Health, New York, NY

^3^Department of Medicine, NYU Grossman School of Medicine, New York, NY

^4^NYC Health + Hospitals, New York, NY

^5^VA New York Harbor Healthcare System, New York, NY

**Supplement Tables**

Supplement Table 1: Characteristics of Patients Who Were Determined Eligible for the Intervention Arm (and were Outreached by CHWs), by Their Enrollment Status

Supplement Table 2: Characteristics of Patients Who Were Enrolled in the Intervention Arm, by their Intake Completion Status

Supplement Table 3: Moderation of Fidelity Measures by PAM Score Among Intervention Patients Completing the Intake Survey

Supplement Table 4: Moderation of Fidelity Measures by Clinical Site* Among Intervention Patients Completing the Intake Survey

**Supplement Table 1:** **Characteristics of Patients Who Were Determined Eligible for the Intervention Arm (and were Outreached by CHWs), by Their Enrollment Status**

|  | **Eligible** | **Not enrolled** | **Enrolled** |
| --- | --- | --- | --- |
|  | **(N=1449)** | **(N=890)** | **N=559** |
| **Gender (Male)** | 881 (60.8%) | 539 (60.6%) | 342 (61.2%) |
| Missing | 2 (0.1%) | 1 (0.1%) | 1 (0.2%) |
| **Language*** |  |  |  |
| English | 1143 (78.9%) | 722 (81.1%) | 421 (75.3%) |
| Spanish | 262 (18.1%) | 140 (15.7%) | 122 (21.8%) |
| Other | 44 (3.0%) | 28 (3.1%) | 16 (2.9%) |
| **Race*** |  |  |  |
| Asian | 67 (4.6%) | 45 (5.1%) | 22 (3.9%) |
| Black | 377 (26.0%) | 197 (22.1%) | 180 (32.2%) |
| White | 269 (18.6%) | 154 (17.3%) | 115 (20.6%) |
| Other | 254 (17.5%) | 134 (15.1%) | 120 (21.5%) |
| Unknown | 482 (33.3%) | 360 (40.4%) | 122 (21.8%) |
| **Ethnicity*** |  |  |  |
| Hispanic or Latino | 432 (29.8%) | 220 (24.7%) | 212 (37.9%) |
| Not Hispanic or Latino | 1009 (69.6%) | 664 (74.6%) | 345 (61.7%) |
| Unknown | 8 (0.6%) | 6 (0.7%) | 2 (0.4%) |
| **Hospital*** |  |  |  |
| Bellevue | 919 (63.4%) | 591 (66.4%) | 328 (58.7%) |
| VA | 530 (36.6%) | 299 (33.6%) | 231 (41.3%) |
| **Age (yrs) at outreach*** |  |  |  |
| Median [Q1, Q3] | 58.3 [47.9, 66.0] | 57.9 [47.2, 65.4] | 58.6 [49.4, 67.1] |
| Missing | 2 (0.1%) | 1 (0.1%) | 1 (0.2%) |
| **Pam scoreᵃ** |  |  |  |
| Median [Q1, Q3] | 18.0 [18.0, 21.0] | NA | 18.0 [18.0, 21.0] |
| Missing | 1037 (71.6%) | NA | 147 (26.3%) |
| ᵃ Only for patients who completed intake; * p<0.001 | | | |

**Supplement Table 2:** **Characteristics of Patients Who Were Enrolled in the Intervention Arm, by their Intake Completion Status**

|  | **Enrolled** | **Intake completed** | **Intake not completed** |
| --- | --- | --- | --- |
|  | **N=559** | **N=444** | **N=115** |
| **Gender (Male)** | 342 (61.2%) | 264 (59.5%) | 78 (67.8%) |
| Missing | 1 (0.2%) | 0 (0%) | 1 (0.9%) |
| **Language** |  |  |  |
| English | 421 (75.3%) | 335 (75.5%) | 86 (74.8%) |
| Spanish | 122 (21.8%) | 97 (21.8%) | 25 (21.7%) |
| Other | 16 (2.9%) | 12 (2.7%) | 4 (3.5%) |
| **Race** |  |  |  |
| Asian | 22 (3.9%) | 18 (4.1%) | 4 (3.5%) |
| Black | 180 (32.2%) | 138 (31.1%) | 42 (36.5%) |
| White | 115 (20.6%) | 92 (20.7%) | 23 (20.0%) |
| Other | 120 (21.5%) | 96 (21.6%) | 24 (20.9%) |
| Unknown | 122 (21.8%) | 100 (22.5%) | 22 (19.1%) |
| **Ethnicity** |  |  |  |
| Hispanic or Latino | 212 (37.9%) | 174 (39.2%) | 38 (33.0%) |
| Not Hispanic or Latino | 345 (61.7%) | 269 (60.6%) | 76 (66.1%) |
| Unknown | 2 (0.4%) | 1 (0.2%) | 1 (0.9%) |
| **Hospital** |  |  |  |
| Bellevue | 328 (58.7%) | 265 (59.7%) | 63 (54.8%) |
| VA | 231 (41.3%) | 179 (40.3%) | 52 (45.2%) |
| **Age (years) at outreach** |  |  |  |
| Median [Q1, Q3] | 58.6 [49.4, 67.1] | 58.7 [49.2, 67.2] | 58.2 [50.2, 66.5] |
| Missing | 1 (0.2%) | 0 (0%) | 1 (0.9%) |
| **Pam scoreᵃ** |  |  |  |
| Median [Q1, Q3] | 18.0 [18.0, 21.0] | 18.0 [18.0, 21.0] | NA |
| Missing | 147 (26.3%) | 32 (7.2%) | 115 (100%) |
| ᵃ Only for patients who completed intake | | | |

**Supplement Table 3: Moderation of Fidelity Measures by PAM Score Among Intervention Patients Completing the Intake Survey**

| **Fidelity Measure** | **PAM score available ^a^**  **N = 412** | **PAM score**  **≤ Median**  **N = 231** | **PAM score**  **> Median**  **N = 181** | **Unadjusted  P-value** | **Adjusted**  **P-value ^b^** |
| --- | --- | --- | --- | --- | --- |
| COVERAGE^c^ |  |  |  |  |  |
| Percent who completed the first core component of establishing at least one goal or a Health Action Plan^d^ | 80.8% | 78.8% | 83.4% | 0.289 | 0.259 |
| CONTENT ADHERENCE |  |  |  |  |  |
| Percent who established at least one goal or a Health Action Plan^d^ | 80.8% | 78.8% | 83.4% | 0.289 | 0.259 |
| Percent who received coaching on least one education topic | 81.8% | 80.1% | 84.0% | 0.375 | 0.411 |
| Percent who received coaching on all education modules | 45.4% | 44.2% | 47.0% | 0.64 | 0.936 |
| Percent who received at least one PC visit | 79.4% | 78.8% | 80.1% | 0.836 | 0.326 |
| Percent who received at least one referral | 43.4% | 47.2% | 38.7% | 0.103 | 0.305 |
| Percent who had at least one successful encounter | 78.6% | 78.4% | 79.0% | 0.969 | 0.395 |
| Percent who received all 4 core components in some capacity | 33.3% | 36.8% | 28.7% | 0.105 | 0.611 |
| DOSE-FREQUENCY |  |  |  |  |  |
| Median [IQR] number of goals established | 2 [2, 3] | 3 [2, 3] | 2 [2, 3] | 0.939 | 0.441 |
| Median [IQR] number of goals completed^e^ | 1 [0, 2] | 1 [0, 2] | 1 [0, 2] | 0.760 | 0.553 |
| Median [IQR] number of education sessions delivered | 17 [7, 31] | 17 [7, 34] | 17 [8, 29] | 0.634 | 0.943 |
| Median [IQR] number of education modules covered | 4 [2, 4] | 4 [2, 4] | 4 [2, 4] | 0.335 | 0.256 |
| Median [IQR] number of PC visits | 3 [2, 5] | 3 [2, 5] | 3 [2, 5] | 0.117 | 0.180 |
| Median [IQR] number of referrals | 1 [1, 2.5] | 1 [1, 2] | 2 [1, 3] | 0.195 | 0.996 |
| Median [IQR] number of successful encounters | 5 [2, 8] | 6 [3, 9] | 4 [2, 7] | 0.119 | 0.801 |
| DOSE-DURATION |  |  |  |  |  |
| Median [IQR] duration (days) of follow-up time | 405 [334, 440] | 398 [335, 439] | 408 [315, 440] | 0.348 | 0.618 |
| All calculations are among subjects with non-missing data on the fidelity measure being reported  ^a^ Among patients who completed intake, 32 patients did not have a PAM score recorded  ^b^ P values are adjusted for gender, language, race, ethnicity and age through multivariate regressions  ^c^ Two coverage measures - Percent of outreached patients who were enrolled and Percent of enrolled patients who completed an intake – were not evaluated for moderation because they do not make up our denominator (number of patients completing intake). These measures have been reported in text and Tables 3 and 4.  ^d^ Percent of intake patients who established at least one goal or completed establishing a Health Action Plan was operationalized as a measure of two fidelity constructs – content adherence and coverage – because this component was the first component that patients were required to complete in order to proceed with other components of the intervention  ^e^ Calculated among subjects who established at least one goal | | | | | |

**Supplement Table 4: Moderation of Fidelity Measures by Clinical Site* Among Intervention Patients Completing the Intake Survey**

| **Fidelity Measure** | **Completed intake**  **(N = 444)** | **Bellevue Hospital**  **(N = 265)** | **VA**  **(N = 179)** | **Unadjusted**  **P-value** | **Adjusted**  **P-value ^a^** |
| --- | --- | --- | --- | --- | --- |
| COVERAGE ^b^ |  |  |  |  |  |
| Percent who completed the first core component (established at least one goal or a Health Action Plan) ^c^ | 81.5% | 77.4% | 87.7% | 0.008 | 0.011 |
| CONTENT ADHERENCE |  |  |  |  |  |
| Percent who established at least one goal or a Health Action Plan ^c^ | 81.5% | 77.4% | 87.7% | 0.008 | 0.011 |
| Percent who received coaching  on least one education topic | 82.7% | 78.9% | 88.3% | 0.015 | 0.014 |
| Percent who received coaching  on all education modules | 40.0% | 58.7% | 47.5% | <0.001 | 0.003 |
| Percent who received  at least one PC visit | 79.5% | 82.3% | 75.4% | 0.102 | 0.134 |
| Percent who received  at least one referral | 45.0% | 47.9% | 40.8% | 0.166 | 0.051 |
| Percent who had at least  one successful encounter | 79.7% | 80.4% | 78.8% | 0.770 | 0.422 |
| Percent who received all 4 core components in some capacity | 34.9% | 41.1% | 25.7% | 0.001 | 0.001 |
| DOSE-FREQUENCY |  |  |  |  |  |
| Median [IQR] number  of goals established | 3 [2, 3] | 2 [2, 2] | 2 [2, 3] | <0.001 | 0.128 |
| Median [IQR] number  of goals completed ^d^ | 1 [0, 3] | 0 [0, 2] | 0 [0, 2] | 0.921 | 0.731 |
| Median [IQR] number of  education sessions delivered | 18 [8, 33] | 15 [7, 31] | 20.5 [9, 34] | <0.001 | 0.061 |
| Median [IQR] number of  education modules covered | 4 [2, 4] | 4 [2, 4] | 4 [2, 4] | 0.002 | 0.020 |
| Median [IQR] number of PC visits | 3 [2, 5] | 3 [2, 5] | 3 [2, 5] | 0.295 | 0.045^e^ |
| Median [IQR] number of referrals | 1 [1, 3] | 2 [1, 2] | 1 [1, 3] | 0.179 | 0.242 |
| Median [IQR] number  of successful encounters | 5 [3, 8] | 6 [3, 10] | 4 [2, 7] | 0.004 | 0.045^e^ |
| DOSE-DURATION |  |  |  |  |  |
| Median [IQR] duration (days)  of follow-up time | 411 [341, 446] | 395 [313, 446] | 427 [375, 446] | 0.011 | 0.473 |
| * All calculations are among subjects with non-missing data on the fidelity measure being reported  ^a^P values are adjusted for gender, language, race, ethnicity and age through multivariate regressions  ^b^ Two coverage measures - Percent of outreached patients who were enrolled and Percent of enrolled patients who completed an intake – were not evaluated for moderation because they do not make up  our denominator (number of patients completing intake). These measures have been reported in text and Tables 3 and 4.  ^c^ Percent of intake patients who established at least one goal or completed establishing a Health Action Plan was operationalized as a measure of two fidelity constructs – content adherence and coverage – because this component was the first component that patients were required to complete in order to proceed with other components of the intervention  ^d^ Calculated among subjects who established at least one goal  ^e^p values compare rank sums which are significantly different but medians are equal | | | | | |
